# Kynurenic Acid in Plasma and Endometrium in Bitches with Pyometra

**DOI:** 10.1007/s10753-012-9527-5

**Published:** 2012-08-23

**Authors:** Roman Dąbrowski, Tomasz Kocki, Marek Szczubiał, Wojciech Dąbrowski, Jolanta Parada-Turska

**Affiliations:** 1 Department and Clinic of Animal Reproduction, Faculty of Veterinary Medicine, University of Life Sciences in Lublin, Głęboka 30, 20-612 Lublin, Poland; 2 Department of Experimental and Clinical Pharmacology, Medical University in Lublin, Jaczewskiego 8, 20-090 Lublin, Poland; 3 Department of Anaesthesiology and Intensive Therapy, Medical University in Lublin, Jaczewskiego 8, 20-954 Lublin, Poland; 4 Department of Rheumatology and Connective Tissue Diseases, Medical University in Lublin, Jaczewskiego 8, 20-954 Lublin, Poland

**Keywords:** bitches, inflammation, kynurenic acid, pyometra

## Abstract

Kynurenic acid (KYNA) is produced enzymatically in humans and animals from kynurenine. Reports concerning changes of kynurenine metabolism during inflammation are available in the literature. Pyometra is a pathological condition characterized by the accumulation of pus in the uterine lumen and bacterial infection. The objective of the study was to compare the serum and endometrial KYNA concentrations in healthy bitches and those with pyometra. KYNA was determined by means of high-performance liquid chromatography with fluorometric detection. The serum content of KYNA in bitches with pyometra was significantly higher than in healthy bitches. The KYNA content in the endometrium of bitches with pyometra was higher, yet the difference was not statistically significant. Our result indicates that determination of KYNA might be a marker of pyometra in bitches.

## INTRODUCTION

Kynurenic acid (KYNA) was first detected in dog’s urine and described in 1853 by Liebig. It is produced in vertebrates along the kynurenine pathway of tryptophan metabolism [1]. KYNA is a final product of the kynurenine transamination [2]. Apart from its action on neuronal glutamatergic receptors [3, 4], KYNA is also a ligand of G protein-coupled receptor 35, which demonstrates a high expression on the immune system and the gastrointestinal tract components [5] as well as a ligand of aromatic hydrocarbon receptor participating in the process of carcinogenesis and mechanisms of immune response modulation [6]. There are premises of KYNA’s significance in various inflammatory diseases [7–17].

Pyometra is one of the most frequently occurring pathological conditions of the reproductive tract in adult and older bitches, in the metestrus phase of the estrous cycle. The disease is characterized by the accumulation of a large amount of pus in the uterine lumen, which, in consequence, leads to absorption of suppurating toxins by blood and systemic disturbances, e.g., kidney and liver damage [18].

Pyometra often constitutes a threat to the bitches’ life, and, for this reason, it should be diagnosed and treated as soon as possible. The primary treatment in a case of pyometra is surgical intervention consisting of the simultaneous removal of both the uterus and ovaries [19].

So far, there are no literature data concerning the determination of KYNA in inflammation of uterus in animals. The objective of the study was to compare the serum and endometrial KYNA concentrations in healthy bitches and those with pyometra.

## MATERIALS AND METHODS

All experimental protocols and procedures were approved by the First Local Ethics Committee in Lublin. The study involved 20 bitches of various breeds and mongrels treated in the Department and Clinic of Animal Reproduction, Veterinary Faculty, University of Life Sciences in Lublin. The animals were divided into two groups. Control group consisted of ten clinically healthy bitches, aged 2–6 years, between the 4–11th weeks after estrous cycle, which underwent ovariohysterectomy on the owners’ request. Study group included ten bitches, aged 5–13 years, diagnosed with pyometra based on the medical history, clinical examination, cytology tests, vaginoscopy, and ultrasound examination as well as hematological and blood biochemistry tests. Bitches in this group were between 4 and 10 weeks after estrous cycle. These animals underwent ovariohysterectomy because of pyometra.

In both groups, the surgical procedures were carried out under general anesthesia using intramuscular ketamine of 5–15 mg/kg b.w. (Narkamon 5 %®, Spofa, Czech Republic), following premedication with subcutaneous atropine of 0.05 mg/kg b.w. (Atropinum sulfuricum®, Polfa, Poland), and intramuscular xylazine of 2 mg/kg b.w. (Rometar 2 %®, Spofa, Czech Republic).

Prior to surgery, the blood was sampled (9 ml) from the saphenous vein of all bitches to sterile centrifugation tubes and silicone vacuette (Greiner Labortechnik GmbH, Austria). After blood centrifugation, the serum was frozen and kept at −80 °C until used.

Immediately after surgery, the uterine endometrium biopsies were taken. The endometrium section, 1.0 × 1.0 cm, was excised from the central right horn of the uterus, placed in the plastic Eppendorf tubes and frozen at −80 °C.

KYNA was investigated according to the method of Turski *et al*. [20] and Shibata [21]. In brief, serum samples were acidified with 1 N HCl and 50 % trichloroacetic acid and then centrifuged. Tissue samples were sonicated (1:5 *wt*/*vol*) in distilled water. The resulting homogenate was centrifuged. The supernatant was acidified with 1 N HCl and 50 % trichloroacetic acid and then centrifuged. Supernatant was applied on cation-exchange resin (Dowex 50 W+, Sigma). Eluted KYNA was subjected to the HPLC (Varian HPLC system, ESA catecholamine HR-80, 3 μm, C_18_ reverse-phase column) and quantified fluorometrically (excitation, 344 nm; emission, 398 nm).

The results are presented as a mean ± standard deviation or median values according to data distribution and result of normality test. Data were analyzed using Student *t* test or Mann–Whitney test. The significance of differences was determined at *p* < 0.05.

## RESULTS

All bitches with pyometra showed reduced appetite, polydipsia, apathy, and enlarged abdominal integuments; 80 % of them developed purulent discharge from reproductive organs. In all bitches, abdominal ultrasound examinations revealed enlarged uterus of a diameter ranging from 2 to 7 cm with a hypoechogenic content.

The mean amount of leucocytes in bitches with pyometra was 27.5 ± 8.0 × 10^9^/l and was statistically significantly higher (*p* < 0.05) compared to the values in healthy bitches (11.4 ± 2.5 × 10^9^/l). The percentage of rod neutrophils noted in bitches suffering from pyometra (5.9 ± 1.5 %) was statistically significantly higher (*p* < 0.05) compared to that of healthy bitches (1.7 ± 0.7 %).

According to the bacteriological tests of the uterine purulent secretions, *Escherichia coli* was identified in 90 % of cases; in the remaining 10 %, *Staphylococcus* spp*.* were detected.

**Fig. 1:**
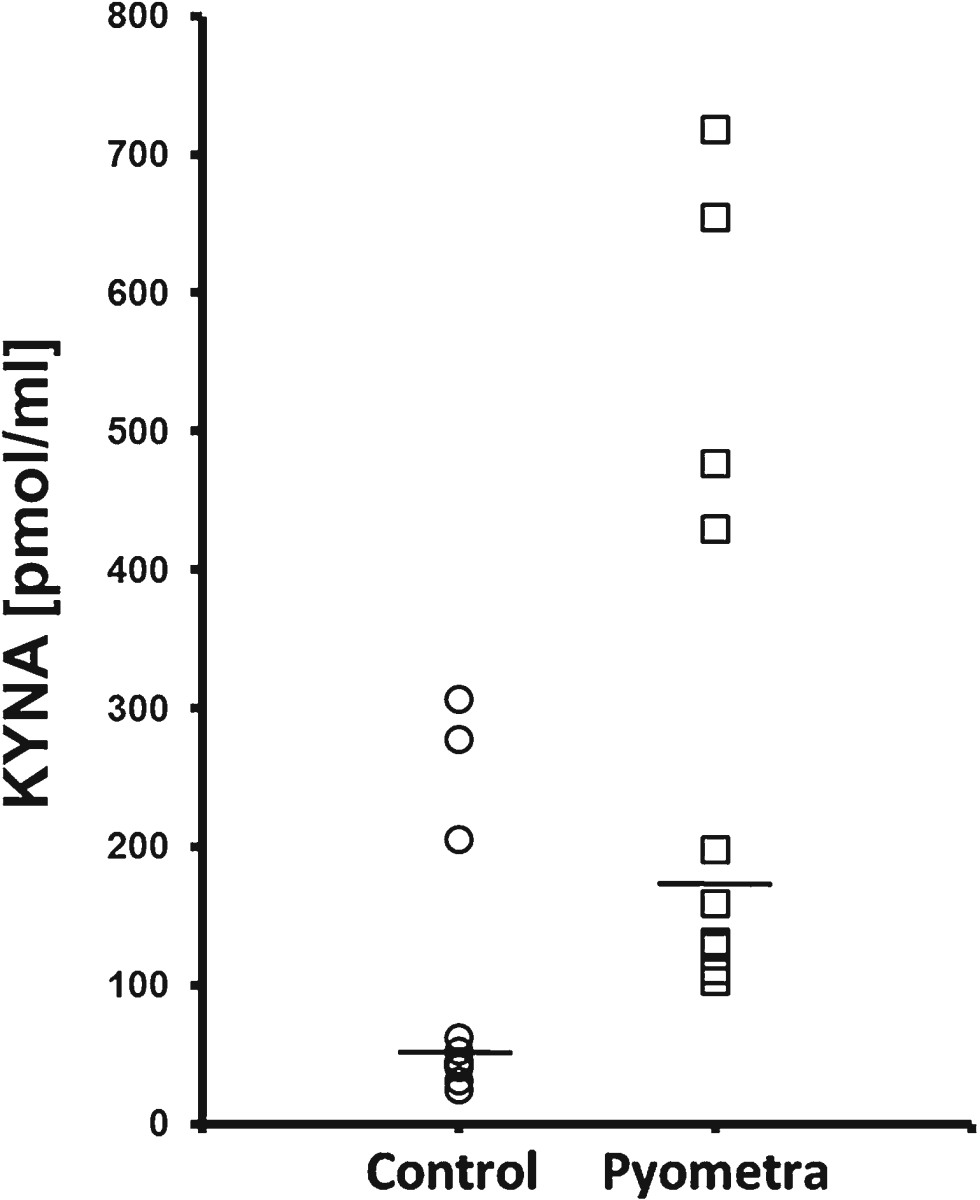
Scatter plots of kynurenic acid (KYNA) concentration in plasma of bitches. Samples were collected from bitches that underwent ovariohysterectomy. Results are expressed as picomoles of KYNA/milliliter. The *horizontal bar* represents the median value.

**Fig. 2:**
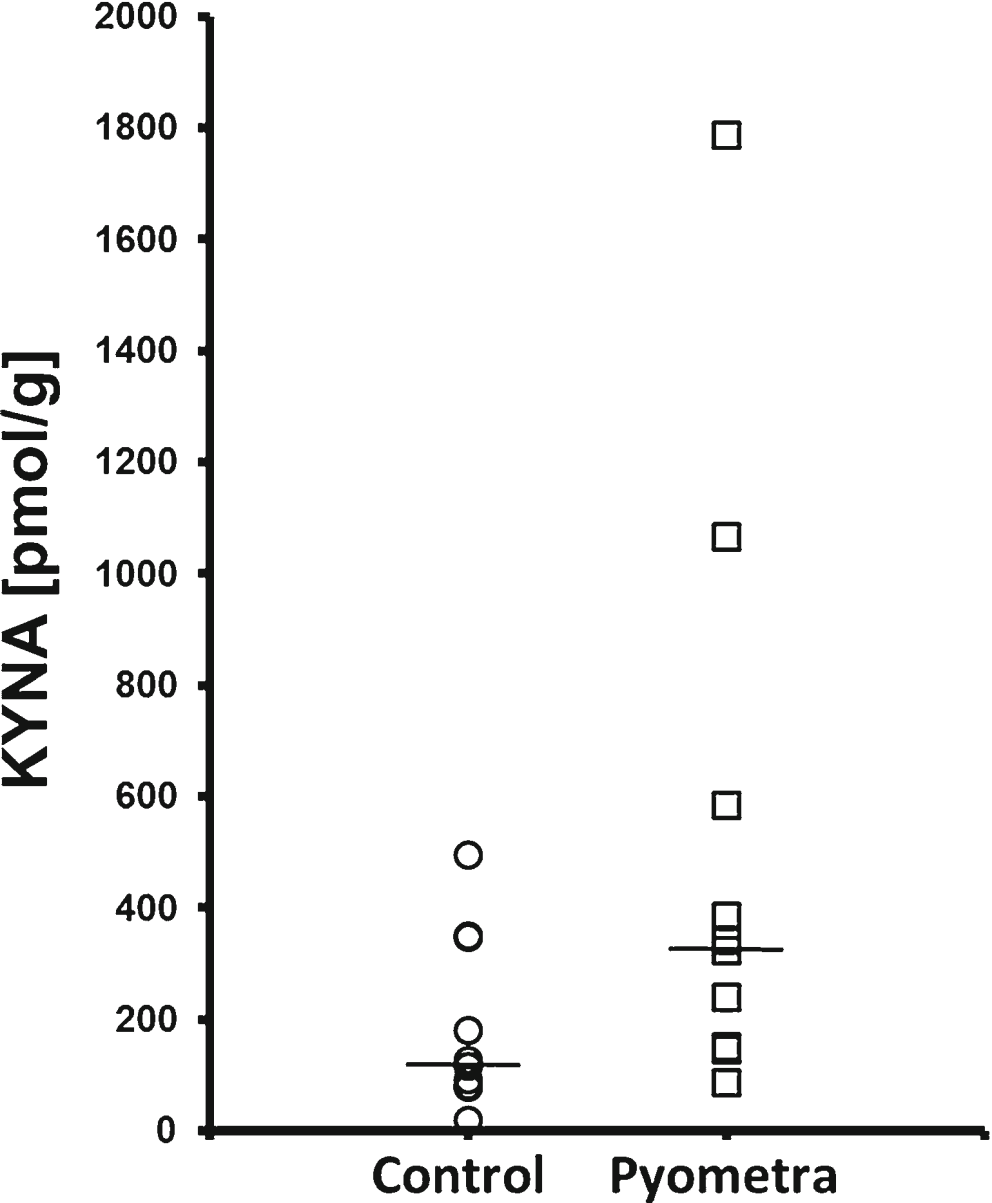
Scatter plots of kynurenic acid (KYNA) concentration in endometrium of bitches. Samples were collected from bitches that underwent ovariohysterectomy. Results are expressed as picomoles of KYNA/gram wet weight. The *horizontal bar* represents the median value.

The median serum content of KYNA in bitches with pyometra and healthy bitches was 179.6 and 49.0 pmol/ml, respectively (Fig. 1). The difference was statistically significant (*p* < 0.05). The median content of KYNA in the endometrium of bitches with pyometra and in healthy animals was 334.1 and 121.5 pmol/g, respectively (Fig. 2). The difference did not reach statistical significance (*p* > 0.05).

Average age of animals with pyometra was 10.8 ± 2.2 years and was significantly higher (*p* < 0.05) compared to that of healthy bitches (3.4 ± 1.4 years). There was no correlation between KYNA content and age in healthy bitches (data not shown).

## DISCUSSION

Results of clinical examination, laboratory, and bacteriological tests confirm the diagnosis of pyometra in the investigated bitches. In bitches with pyometra, a significant increase of blood KYNA concentration has been demonstrated. Our result is consistent with other reports. An increase of KYNA in serum was reported in generalized sepsis in humans [7] and in patients with chronic kidney disease [8]. A significant increase of KYNA in patients with meningitis, septicemia and various autoimmunological diseases as well as with bacterial, viral, fungal, and parasitic infections was demonstrated in the cerebrospinal fluid and in the brain [9]. Similarly, an increase in KYNA concentration in the brain of mice infected with the flu virus has been observed [15]. The increase in KYNA concentration in the spinal cord has been demonstrated in the model of allergic encephalitis and myelitis in rats [16]. Elevated KYNA concentration in the saliva of the patients with odontogenic abscesses has also been found [17]. In our study, a tendency toward an increase of KYNA concentration was found in the endometrium of bitches with pyometra; however, the difference did not reach statistical significance probably due to high variability between the results and limited number of investigated subjects.

It is known that frequency of pyometra increases with the age of animals [22]. In our study, the animals diagnosed with pyometra were significantly older than the control ones. It has been described that in 18-month old rats, the KYNA blood concentration is higher than in 3-month old rats [23]. In contrast, no correlation between the KYNA blood concentration and age in humans has been observed [24]. We found no correlation between the age and KYNA blood concentration in bitches.

KYNA is produced from kynurenine, and the rate of its synthesis depends mainly on precursor availability. Kynurenine is converted from tryptophan by indoleamine 2,3-diooxygenase, which activity is stimulated in the course of inflammatory processes [25, 26]. Moreover, it was found that lipopolysaccharide enhances KYNA production in human gingival fibroblasts [17]. Thus, it can be assumed that an increase in KYNA content is a result of inflammatory response.

Interestingly, data from animal studies indicate that an administration of exogenous KYNA results in anti-inflammatory action. Glavin *et al*. demonstrated that KYNA attenuates experimental gastric ulcer formation produced by ethanol, stress, or toxins derived from the Atlantic coast mussels [27–29]. Kaszaki *et al*. found out that KYNA alleviates the inflammation resulting from experimental colon obstruction in dogs [30]. KYNA restored the tone of the colon and decreased motility index of the giant colonic contractions. It has been demonstrated that KYNA treatment significantly suppressed the increase in plasma nitrite/nitrate (NO_x_) and reduced elevated activity of xanthine oxidase and xanthine dehydrogenase [30]. Similarly, KYNA decreased the motility and increased the tone of the colon in the acute ulcerative colitis model. Moreover, it reduced elevated activity of xanthine oxidoreductase, nitric oxide synthase, and myeloperoxidase [31]. In septicemia induced by the administration of lipopolysaccharide in mice, KYNA significantly reduced the mortality rate of the animals and inhibited nitric oxide, as well as increased tumor necrosis factor α concentration [32].

Based on our results, a suggestion that KYNA concentration measurement might be an inflammation indicator of pyometra in bitches seems justified. It is also possible to call for application of KYNA in the treatment of pyometra, which requires confirmation in further experimental studies.
